# Structural reassignment of compound 968, an allosteric glutaminase inhibitor

**DOI:** 10.3762/bjoc.22.33

**Published:** 2026-03-13

**Authors:** Lindsey A Albertelli, Sainabou Jallow, Chun Li, Scott M Ulrich

**Affiliations:** 1 Department of Chemistry, Ithaca College, Ithaca, NY 14850, USA

**Keywords:** cancer metabolism, compound 968, glutaminase

## Abstract

Many cancer cells require extracellular glutamine to meet the energetic, biosynthetic, and redox demands of the proliferative state. Glutaminases catalyze the hydrolysis of glutamine to glutamate, which supports the biosynthesis of amino acids, lipids, and glutathione and can also be oxidatively deaminated to α-ketoglutarate and enter the citric acid cycle. The “glutamine addiction” of cancer cells has made glutaminase an attractive anticancer drug target. Compound 968 is a glutaminase inhibitor that is widely used to probe cancer cells’ dependence on glutaminase activity. Here, we show by NMR spectroscopy and X-ray crystallography that the reported benzo[*c*]phenanthridine structure of compound 968 is incorrect; its true structure is the isomeric benzo[*c*]acridine. The structural reassignment of compound 968 will aid the medicinal chemistry development of this important compound.

## Introduction

Cancer cells often show a strong reliance on glutamine uptake and metabolism [1–2]. Glutamine serves as a nitrogen donor for the biosynthesis of asparagine, amino sugars, and nucleotides. Glutamine is also hydrolyzed to glutamate by glutaminase (GLS) enzymes, which are often overexpressed in cancer cells. The resulting glutamate supports the biosynthesis of glutathione and can be oxidatively deaminated to the TCA cycle intermediate α-ketoglutarate for energy production and additional biosynthetic pathways. There are two glutaminase isozymes in humans: KGA is encoded by the *GLS1* gene and is expressed mainly in the kidney and brain and LGA encoded by the *GLS2* gene is expressed mainly in the liver. GAC is a highly active splice variant of KGA often expressed in cancer cells [3]. GLS1 and GLS2 enzymes share similar sequences, tetrameric structures, and enzymatic stimulation by phosphate [4–6].

**Figure 1:**
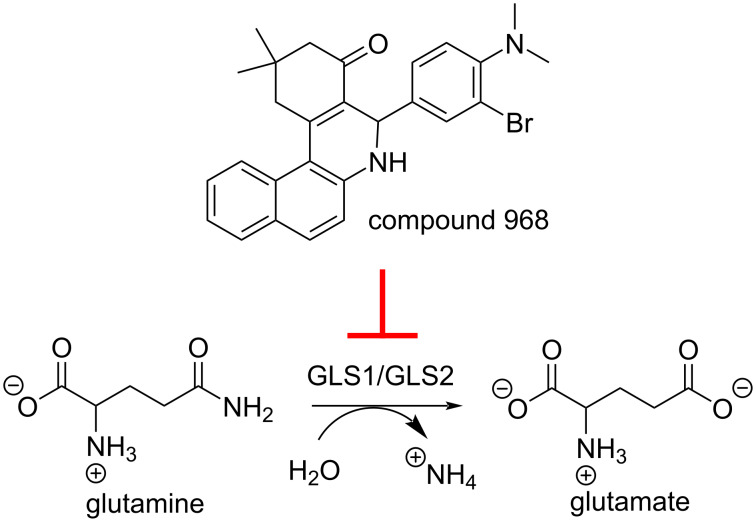
The glutaminase enzymes GLS1 and GLS2 catalyze the hydrolysis of glutamine to glutamate and ammonia. Compound 968 is a dual inhibitor of GLS1 and GLS2.

The reliance of cancer cells on glutamine to meet the energetic, biosynthetic, and redox demands of the proliferative state creates an opportunity to selectively target cancer cells [1,7]. Chemical inhibition of GLS has emerged as an attractive anticancer strategy, and several classes of GLS inhibitors have been discovered [1–2]. DON is a diazo-containing electrophilic glutamine analog that inhibits glutaminase by covalently labeling the catalytic serine [8]. DON inhibits other glutamine-utilizing enzymes such as asparagine synthetase and has activity against a wide range of cancer cell lines [9–10]. BPTES is a potent allosteric inhibitor of GAC/KGA that binds at the dimer–dimer interface and stabilizes a catalytically inactive form of the enzyme [11–12]. The BPTES scaffold has undergone significant medicinal chemistry development resulting in CB-839/telaglenastat, which is being evaluated in clinical trials in combination with other anticancer agents [13]. Compound 968 is a third class of glutaminase inhibitor discovered in a screen for compounds able to revert the transformed phenotype of cells harboring oncogenic Dbl, the nucleotide exchange factor of the small GTPase Rho [14]. Compound 968 is a dual GLS1/GLS2 inhibitor and has found wide use to probe the effects of glutaminase inhibition on several cancer cell lines (Figure 1) [15–16]. The anticancer effects of compound 968 have been tested in combination with other drugs such as paclitaxel [17], erlotinib [18], apigenin [19], metformin [20], and inhibitors of tissue transglutaminase [21]. Compound 968 was recently found to suppress the growth of a luminal breast cancer cell line that expresses GLS2 and is insensitive to GLS1-specific inhibitors such as BPTES and CB-839 [22].

**Figure 2:**
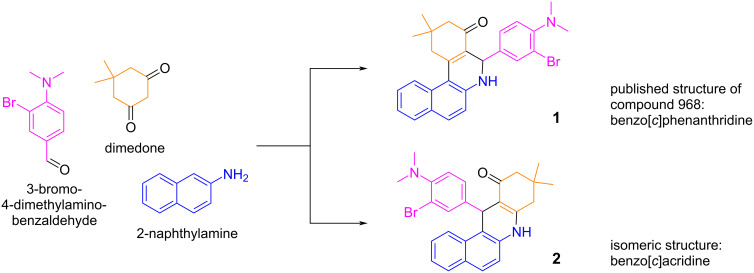
The cycloaddition reaction between an arylaldehyde (shown here is the specific aldehyde that produces compound 968), dimedone, and 2-naphthylamine was originally reported to produce benzo[*c*]phenanthridine **1**, of which the commonly accepted structure of 968 is a member. The structure of the product of this reaction was subsequently reported to be the isomeric benzo[*c*]acridine **2**.

After initiating a project to synthesize derivatives of compound 968, we searched the literature for synthetic routes towards its scaffold. Compound 968 is a benzo[*c*]phenanthridine, made by a three-component cyclocondensation reaction between the 1,3 diketone dimedone, the aryl aldehyde 3-bromo-4-dimethylaminobenzaldehyde, and 2-naphthylamine. This reaction producing the benzo[*c*]phenanthridine core was first reported in the late 1960s [23–24] and the substrate scope of diketone, arylamine, and arylaldehyde was widened over the subsequent decades [25–27]. However, later reports by Martinez et al. [28] using X-ray crystallography and Kozlov et al. [29–30] using NOESY NMR showed that this three-component cyclocondensation reaction does not produce benzo[*c*]phenanthridine **1** as product, but instead yields the isomeric benzo[*c*]acridine **2** (Figure 2).

Upon learning of these results, we became concerned that the accepted structure of compound 968 is incorrect. We then sought to determine whether compound 968 is a benzo[*c*]phenanthridine **1** or benzo[*c*]acridine **2**. Clarifying this issue would benefit the community of cancer biologists who use compound 968, enable medicinal chemistry around the compound 968 scaffold, as well as correct the structure displayed by vendors of this compound.

## Results and Discussion

To determine whether the structure of compound 968 is a benzo[*c*]phenanthridine **1** or benzo[*c*]acridine **2** we synthesized it by the reported cyclocondensation reaction, which consists of heating an equimolar mixture of 3-bromo-4-dimethylaminobenzaldehyde, 2-naphthylamine, and dimedone to reflux in 1-butanol. We also purchased compound 968 from Sigma (catalog # 352010; CAS # 311795-38-7) which is advertised as the benzo[*c*]phenanthridine isomer **1**. We also purchased material advertised as benzo[*c*]acridine isomer **2**, which is available from ChemDiv (catalog # 8012-8736; CAS # 442660-67-5). Based on the results of Martinez and Kozlov reassigning the structure of the cycloaddition reaction product from benzo[*c*]phenanthridine **1** to benzo[*c*]acridine **2**, we predicted that all three compounds are the benzo[*c*]acridine isomer **2**.

**Figure 3:**
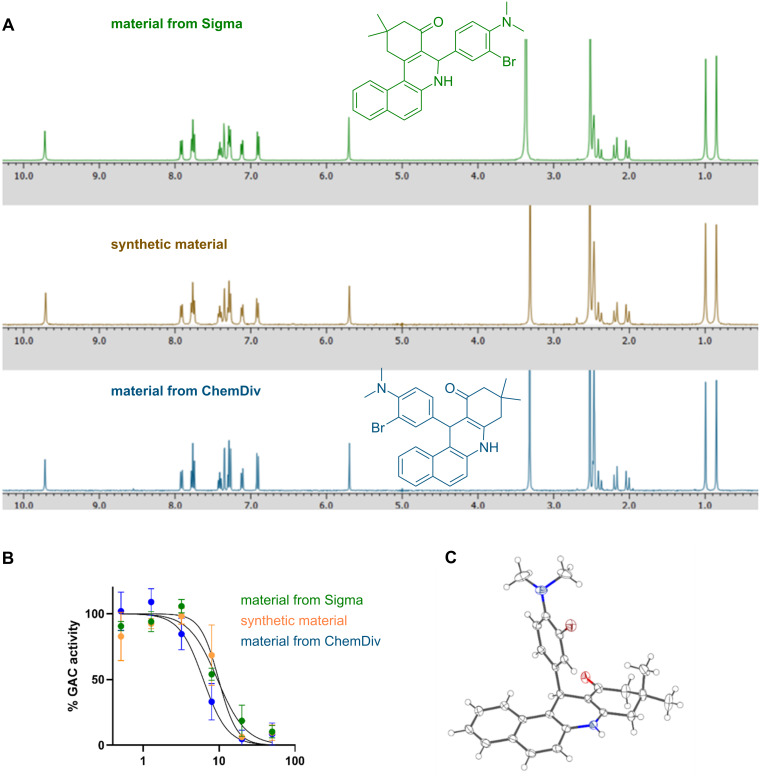
A) ^1^H NMR spectra of compound 968 purchased from Sigma-Aldrich (top), the same compound synthesized using the published cyclocondensation reaction (middle), and the isomeric benzo[*c*]acridine compound purchased from ChemDiv. B) Dose–response curves of the three compounds against the glutaminase isoform GAC. C) ORTEP diagram of the X-ray crystal structure of the material synthesized according to the published cyclocondensation reaction.

The ^1^H NMR spectra of all three compounds are identical, indicating that they share the same structure (Figure 3A). We assayed each compound for inhibitory activity against the GAC isoform of glutaminase and found that the three compounds inhibited it with equal potency, further suggesting that the compounds are identical (Figure 3B). To unambiguously determine the structure of this compound, we grew crystals of our synthetic material and solved the structure by X-ray crystallography. The structure that fits the diffraction data is the benzo[*c*]acridine isomer **2** (Figure 3C). From this, we conclude that the structure of compound 968 is not the commonly accepted benzo[*c*]phenanthridine **1** but is instead the benzo[*c*]acridine isomer **2** in agreement with the reports of Martinez and Koslov. Since the only reaction reported to give the benzo[*c*]phenanthridine isomer **1** is the three-component reaction that instead produces the isomeric benzo[*c*]acridine **2**, we believe that the synthesis of benzo[*c*]phenanthridine isomer **1** remains unrealized.

**Figure 4:**
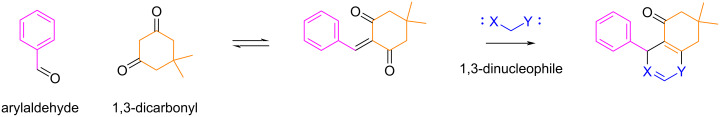
The cyclocondensation reaction that produces compound 968 and related compounds.

This result places the cyclocondensation reaction that generates compound 968 within a family of three-component reactions of 1,3 dicarbonyl compounds, an aryl aldehyde, and various 1,3 di-nucleophiles (Figure 4). These reactions proceed by an aldol condensation between the dicarbonyl and aldehyde, followed by conjugate addition and cyclo-condensation with a 1,3 dinucleophile. Members of this reaction family are variations on the Biginelli and Hantzch reactions, where the dinucleophile is urea and the ammonia adduct of a second equivalent of the dicarbonyl, respectively [31]. A similar reaction with 2-naphthol as the dinucleophile is also known [32]. In all cases, the regiochemistry of cyclization is selective and no isomeric products are formed.

## Conclusion

In this report, we confirm the finding by Martinez [28] and Kozlov [29–30] that the three-component reaction originally claimed to generate benzo[*c*]phenanthridines [23–24] instead generates the isomeric benzo[*c*]acridines and show that this correction applies to compound 968, a prominent member of this class. The compound library that 968 originated from may have been structurally annotated before the reports of Martinez and Koslov were published or without knowledge of these results. The structure reassignment of compound 968 should aid in medicinal chemistry efforts around this scaffold with increased potency and improved pharmacological properties.

## Experimental

### General

X-ray data were obtained on a Bruker Smart Breeze CCD diffractometer. Nuclear magnetic resonance spectra were recorded on a JEOL ECX400 spectrometer. Glutaminase assays were measured on a Tecan Infinite M nano spectrophotometer. Reagents and solvents for chemical synthesis were used without additional purification.

### Chemical synthesis

#### 3-Bromo-4-dimethylaminobenzaldehyde

4-Dimethylaminobenzaldehyde (4.02 g, 26.9 mmol) was dissolved in 1,4-dioxane (52 mL) and NBS (5.01 g, 28.2 mmol) was added in small portions with stirring over 5 minutes. The reaction mixture was stirred at room temperature for 20 minutes then poured into 50 mL water. The mixture was diluted with EtOAc (70 mL), which was separated and washed with water (3 × 50 mL). The organic layer was dried with anhydrous MgSO_4_, filtered, and the solvent removed by rotary evaporation to yield 3-bromo-4-dimethylaminobenzaldehyde as a thick gold oil (5.49 g, 89%). ^1^H NMR (400 MHz, CDCl_3_) δ 9.78 (s, 1H), 8.01 (m, 1H), 7.71 (dd, *J* = 8.7, 2.3 Hz, 1H), 7.04 (d, *J* = 8.7 Hz, 1H), 2.93 (s, 6H); ^13^C NMR (100 MHz, CDCl_3_) δ 189.7, 156.9, 136.1, 131.1, 130.0, 119.5, 116.7, 43.6.

#### 12-(3-Bromo-4-(dimethylamino)phenyl)-8,9,10,12-tetrahydro-9,9-dimethylbenzo[*a*]acridin-11(7*H*)-one (**2**); suggested revised structure for compound 968

3-Bromo-4-dimethylaminobenzaldehyde (2.71 g, 11.8 mmol), 2-naphthylamine (1.70 g, 11.8 mmol), and dimedone (1.66 g, 11.8 mmol) were dissolved in 1-butanol (40 mL) and heated to reflux for 3 hours, during which time a precipitate formed. This precipitate was collected by filtration and washed with ethanol to yield the title compound as a light gray powder (4.05 g, 72%). ^1^H NMR (400 MHz, DMSO-*d*_6_) δ 9.72 (s, 1H), 7.91 (d, *J* = 8.7 Hz, 2H), 7.76 (t, *J* = 6.9 Hz, 2H), 7.41 (t, *J* = 7.3 Hz, 1H), 7.35 (d, *J* = 1.8 Hz, 1H), 7.29 (m, 2H), 7.71 (dd, *J* = 8.3, 1.8 Hz, 1H), 6.90 (d, *J* = 8.2 Hz, 1H), 5.70 (s, 1H), 2.51 (s, 6H), 2.39 (d, *J* = 16.5 H, 1Hz), 2.18 (d, *J* = 16.0 Hz, 1H), 2.02 (d, *J* = 16.0 Hz, 1H), 0.99 (s, 3H), 0.85 (s, 3H). *One diastereotopic methylene hydrogen is obscured by the residual solvent peak at 2.47 ppm; *^13^C NMR (100 MHz, DMSO-*d*_6_) δ 193.72, 151.37, 149.50, 143.73, 134.80, 132.80, 131.69, 130.92, 129.02, 128.68, 128.12, 127.49, 124.25, 122.93, 120.88, 118.45, 117.59, 116.53, 107.43, 50.77, 44.22, 35.30, 32.80, 29.56, 27.06.

#### X-ray crystallography

Crystals of compound **2** were grown by slow evaporation from a saturated acetone solution. A yellow, multi-faceted block of suitable size (0.362 × 0.124 × 0.064 mm^3^) and quality was selected from a representative sample of crystals of the same habit using an optical microscope, mounted onto a Mitegen MicroLoops^TM^ (MiTeGen, LLC., Ithaca, NY) and placed in a cold nitrogen stream of nitrogen. Low temperature (100 K) X-ray data were obtained on a Bruker Smart Breeze CCD diffractometer (Mo sealed X-ray tube, Kα = 0.71073 Å). All diffractometer manipulations, including data collection, integration and scaling were carried out using the Bruker APEXII software. The structure was solved using SHELXS [33] and was refined using SHELXL [34]. Olex2 was employed for the final data presentation and structure plots [35].

The crystal data have been deposited in the Cambridge Crystallographic Data Centre, deposition number CCDC 2426481.

### Enzymatic assay

#### Expression and purification of the GAC isoform of GLS1

Overnight cultures of BL21(DE3) strains carrying GAC cloned into pQE80 were grown at 37 °C overnight in LB supplemented with 100 µg/mL ampicillin. These cultures were diluted 1:100 into 2 L of LB media supplemented with 100 µg/mL ampicillin and grown at 37 °C with shaking at 225 rpm until the OD_600_ reached 0.6. IPTG was added to a final concentration of 0.5 mM and the cultures were shaken at 17 °C for 18 hours. The cells were harvested by centrifugation and stored at −80 °C. The cell pellets were lysed in 20 mL B-PER complete (ThermoFisher) supplemented with an EDTA-free protease inhibitor tablet (Roche) according to the manufacturer’s instructions. The supernatant was applied to 2 mL of Ni:NTA resin (Thermo Scientific) equilibrated with wash buffer (50 mM Tris (pH 8.0), 500 mM NaCl) and rocked at 4 °C for 30 minutes. The beads were transferred to a disposable column and drained. The bead bed was washed at 4 °C with 30 mL of wash buffer and 20 mL of wash buffer supplemented with 10 mM imidazole. Protein was eluted into 1 mL fractions at 4 °C with wash buffer supplemented with 300 mM imidazole. A 20 µL aliquot of each fraction was analyzed by SDS-PAGE. Fractions with pure protein were pooled and the buffer was changed to 20 mM Tris (pH 8.5), 120 mM NaCl using a PD-10 desalting column (Cytiva). The eluent was divided into aliquots, snap frozen in liquid nitrogen, and stored at −80 °C.

#### Glutaminase enzymatic assay

The GAC isoform of glutaminase (79 µL, 50 nM) in Tris acetate (65 mM, pH 8.6) was added to flat, clear-bottomed 96 well plates. A solution of the test compound in DMSO or DMSO itself was added (1 µL), mixed by gently pipetting up and down, then incubated for seven minutes at room temperature. The glutaminase reaction was initiated by addition of 20 µL of a solution of glutamine (100 mM) and K_2_HPO_4_ (500 mM), then mixed by gently pipetting up and down and incubated at room temperature for seven minutes. The reactions were quenched by addition of cold 3 M HCl (10 µL). An aliquot (10 µL) of each quenched GAC reaction was added to 190 µL of a glutamate dehydrogenase reaction which consisted of a solution containing Tris-HCl (100 mM, pH 9.4), NAD^+^ (2 mM), glutamate dehydrogenase (1 µL of a 50% glycerol solution, 15 mg/mL), and hydrazine (0.5 µL) and incubated at room temperature for 40 min. The absorbance at 340 nm was measured and converted to glutamate concentration using the extinction coefficient for NADH of 6220 M^−1^ cm^−1^.

## Data Availability

All data that supports the findings of this study is available in the published article and/or the supporting information of this article. The crystal data have been deposited in the Cambridge Crystallographic Data Centre (https://www.ccdc.cam.ac.uk/) deposition number CCDC 2426481.

## References

[1] Hensley C T, Wasti A T, DeBerardinis R J (2013). J Clin Invest.

[2] Yang W-H, Qiu Y, Stamatatos O, Janowitz T, Lukey M J (2021). Trends Cancer.

[3] van den Heuvel A P J, Jing J, Wooster R F, Bachman K E (2012). Cancer Biol Ther.

[4] Li Y, Erickson J W, Stalnecker C A, Katt W P, Huang Q, Cerione R A, Ramachandran S (2016). J Biol Chem.

[5] Ferreira I M, Quesñay J E N, Bastos A C S, Rodrigues C T, Vollmar M, Krojer T, Strain-Damerell C, Burgess-Brown N A, von Delft F, Yue W W (2021). Biochimie.

[6] Nguyen T-T T, Ramachandran S, Hill M J, Cerione R A (2022). J Biol Chem.

[7] Jin J, Byun J-K, Choi Y-K, Park K-G (2023). Exp Mol Med.

[8] Thangavelu K, Chong Q Y, Low B C, Sivaraman J (2014). Sci Rep.

[9] Novotná K, Tenora L, Slusher B S, Rais R (2024). Adv Pharmacol (San Diego, CA, U S).

[10] Recouvreux M V, Grenier S F, Zhang Y, Esparza E, Lambies G, Galapate C M, Maganti S, Duong-Polk K, Bhullar D, Naeem R (2023). Nat Cancer.

[11] Thangavelu K, Pan C Q, Karlberg T, Balaji G, Uttamchandani M, Suresh V, Schüler H, Low B C, Sivaraman J (2012). Proc Natl Acad Sci U S A.

[12] Robinson M M, Mcbryant S J, Tsukamoto T, Rojas C, Ferraris D V, Hamilton S K, Hansen J C, Curthoys N P (2007). Biochem J.

[13] (2026). Telaglenastat, CB-839·HCl; ClinicalTrials.gov; National Center for Biotechnology Information; National Library of Medicine.

[14] Wang J-B, Erickson J W, Fuji R, Ramachandran S, Gao P, Dinavahi R, Wilson K F, Ambrosio A L B, Dias S M G, Dang C V (2010). Cancer Cell.

[15] Xi J, Sun Y, Zhang M, Fa Z, Wan Y, Min Z, Xu H, Xu C, Tang J (2019). Exp Cell Res.

[16] Han T, Guo M, Zhang T, Gan M, Xie C, Wang J-B (2017). Oncotarget.

[17] Yuan L, Sheng X, Clark L H, Zhang L, Guo H, Jones H M, Willson A K, Gehrig P A, Zhou C, Bae-Jump V L (2016). Am J Transl Res.

[18] Xie C, Jin J, Bao X, Zhan W-H, Han T-Y, Gan M, Zhang C, Wang J (2016). Oncotarget.

[19] Lee Y-M, Lee G, Oh T-I, Kim B M, Shim D-W, Lee K-H, Kim Y J, Lim B O, Lim J-H (2016). Int J Oncol.

[20] Kim J H, Lee K J, Seo Y, Kwon J-H, Yoon J P, Kang J Y, Lee H J, Park S J, Hong S P, Cheon J H (2018). Sci Rep.

[21] Katt W P, Antonyak M A, Cerione R A (2015). Mol Pharmaceutics.

[22] Lukey M J, Cluntun A A, Katt W P, Lin M-c J, Druso J E, Ramachandran S, Erickson J W, Le H H, Wang Z-E, Blank B (2019). Cell Rep.

[23] Lielbriedis I E, Chirkova V V, Gudrinietse E (1969). Izv Akad Nauk Latv SSR, Ser Khim.

[24] Kozlov N S, Nugumanov Z Z (1968). Izv Akad Nauk BSSR, Ser Khim Nauk.

[25] Gusak K N, Tereshko A B, Kozlov N G, Shakailo N I (2000). Russ J Gen Chem.

[26] Kozlov N G, Gusak K N (1999). Russ J Org Chem.

[27] Kozlov N G, Gusak K N, Bezborodov V S (2000). Russ J Org Chem.

[28] Martínez R, Cortés E, Toscano R A, Linzaga I (1990). J Heterocycl Chem.

[29] Kozlov N G, Gusak K N, Tkachev A V (2007). Chem Heterocycl Compd.

[30] Kozlov N G, Gusak K N (2006). Russ J Gen Chem.

[31] Nikoofar K, Yielzoleh F M (2018). J Saudi Chem Soc.

[32] Das B, Laxminarayana K, Krishnaiah M, Srinivas Y (2007). Synlett.

[33] Sheldrick G M (2008). Acta Crystallogr, Sect A: Found Crystallogr.

[34] Sheldrick G M (2015). Acta Crystallogr, Sect C: Struct Chem.

[35] Dolomanov O V, Bourhis L J, Gildea R J, Howard J A K, Puschmann H (2009). J Appl Crystallogr.

